# Mechanism Underlying Time-dependent Cross-phenomenon between Concentration-response Curves and Concentration Addition Curves: A Case Study of Sulfonamides-Erythromycin mixtures on *Escherichia coli*

**DOI:** 10.1038/srep33718

**Published:** 2016-09-20

**Authors:** Haoyu Sun, Hongming Ge, Min Zheng, Zhifen Lin, Ying Liu

**Affiliations:** 1 State Key Laboratory of Pollution Control and Resource Reuse, College of Environmental Science and Engineering, Tongji University, Shanghai 200092, China; 2 Collaborative Innovation Center for Regional Environmental Quality, China; 3 Shanghai Key Laboratory of Chemical Assessment and Sustainability, Shanghai, China

## Abstract

Previous studies have identified a phenomenon in which the concentration-response curves (CRCs) for mixtures cross the curves for concentration addition model when predicting or judging joint toxic actions. However, mechanistic investigations of this phenomenon are extremely limited. In this study, a similar phenomenon was observed when we determined the joint toxic actions of sulfonamides (SAs) and erythromycin (ERY) on *Escherichia coli* (*E*. *coli*), which we named the “cross-phenomenon”, and it was characterized by antagonism in the low-concentration range, addition in the medium-concentration range, and synergism in the high-concentration range. The mechanistic investigation of the cross-phenomenon was as follows: SAs and ERY could form a double block to inhibit the bacterial growth by exhibiting a synergistic effect; however, the hormetic effect of SAs on *E*. *coli* led to antagonism in the low-concentration range, resulting from the stimulation of *sdiA* mRNA expression by SAs, which increased the expression of the efflux pump (AcrAB-TolC) to discharge ERY. Furthermore, this cross-phenomenon was observed to be a time-dependent process induced by the increase of both the concentration and extent of stimulation of *sdiA* mRNA with exposure time. This work explains the dose-dependent and time-dependent cross-phenomenon and provides evidence regarding the interaction between hormesis and cross-phenomenon.

Indiscriminate use of chemicals and ineffective supervision have led to human beings to become exposed to an environment that contains complicated chemical mixtures rather than single chemicals, some of which seriously threatens human health and economic development^1^,^2^. Although the toxicity of many single chemicals has been tested and evaluated to determine their potential for harm and propose safety measures, toxicological prediction and evaluation of the joint effects of chemical mixtures remain a challenge in environmental toxicology^3^,^4^.

In 1939, Bliss first divided joint toxic actions into synergism, antagonism and addition when analyzing the joint toxicity of poisons^5^. Subsequently, some reference indexes have been applied to predict and judge the joint toxic actions of mixtures, such as toxic unit (TU), additive index, mixture toxicity index and so on^6^,^7^,^8^. With the development of investigations into the joint toxicity of chemical mixtures, some reference models, especially the concentration addition (CA) model, have been explored and introduced to this field based on its wide concentration range for comparing actual concentration-response curves (CRCs) with the curves of reference models^9^,^10^. When using the CA model to predict or judge joint toxic action, some studies have found that the joint toxic action of several mixtures varied with the dose in a given organism^11^,^12^,^13^,^14^,^15^, which is different from the results obtained using toxic indexes such as TU, because these toxic indexes only determine the joint toxic action of combined chemicals at a fixed concentration point that is always set at median effective concentration (EC_50_), indicating a fixed joint toxic action (see Figure S1).

The heterogeneous pattern of joint toxic action mentioned above has been identified by previous studies. For example, the determination of the joint toxicity of ionic liquids (1-butyl–2, 3-dimethylimidazolium chloride and 1-butyl-pyridinium bromide) and pesticides (desmetryn and dichlorvos) showed that all the binary mixtures exhibited a similar toxicity action rule, i.e., they displayed a synergistic interaction in the high-concentration range, an additive interaction in the mid-concentration range, and an antagonistic interaction in the low-concentration range^14^. Furthermore, the combined toxicities of erythromycin with levofloxacin and tetracycline to the cyanobacterium *Anabaena* CPB4337 and the green alga *Pseudokirchneriella subcapitata* also showed that the nature of the joint toxic action changed with the concentrations of the chemical mixture^15^. Although this phenomenon has been observed and confirmed, mechanistic investigation of this phenomenon remains extremely limited. A similar phenomenon was observed in our preliminary experiment in which the CRCs for the joint effects of sulfachloropyridazine (SCP) and erythromycin (ERY) on *Escherichia coli* (*E*. *coli*) crossed the curves for the CA model, leading to a heterogeneous pattern of joint toxic action, which we named the “cross-phenomenon”. This observation raises an important issue that we will examine in the current study: why do joint toxic actions vary with the dose of a mixture?

Toxic effects that change with the dose of an agent can also be observed in hormesis of single chemicals, which is characterized by low-dose stimulation and high-dose inhibition^16^. Hormesis has been reported in many studies using a broad range of organisms and across a wide range of chemicals, which gives evidence to prove the universality of hormesis^17^. Using molecular docking studies of the LuxR protein and sulfonamides (SAs), the hormetic effect of SAs on *Photobacterium phosphoreum* was investigated in our previous study, and the mechanistic hypothesis could be described briefly as follows: SAs at low concentrations could bind to the LuxR protein, and the resulting complexes promoted the expression of the LuxR protein^18^.

Because SAs and ERY have been widely used in medicine and pharmacology^19^,^20^, and their toxicities have caused wide concern because of their frequent detection in the environment, such as in water and soil^21^,^22^,^23^,^24^, they were selected as test chemicals in this study, for which the purpose is as follows: first, to determine the toxic effects of binary mixtures of SAs and ERY on *E*. *coli* for 0–24 hours; second, to judge the joint toxic actions of these mixtures using the CA model; third, to investigate the mechanism of the cross-phenomenon through molecular docking studies and by measuring the expression of *sdiA* mRNA in *E*. *coli*; and fourth, to clarify the reason why the cross-phenomenon varies with increasing exposure time.

## Results

### The joint toxicities of SAs and ERY to *E*. *coli*

Before determining their joint toxicities, the individual toxic effects of SAs and ERY on *E*. *coli* were tested after the bacteria were cultured for 12 hours, and the relative CRCs are shown in Figure S2. Based on the EC_50_ values (Table 1) calculated from the CRCs, the order of toxicity of the test chemicals to *E*. *coli* at 12 h was as follows: ERY > Sulfamonomethoxine (SMM) > Sulfaquinoxaline SQ) > Sulfamerazine (SMR) > Sulfameter (SM) > Sulfisoxazole (SSZ) > Sulfadiazine (SD) > Sulfachloropyridazine (SCP) > Sulfadoxine (SDX) > Sulfamethazine (SMZ) > Sulfapyridine (SPY) > Sulfamethoxypyridazine (SMP), and the toxicity of ERY was clearly higher than the toxicities of the SAs.

**Figure 1:**
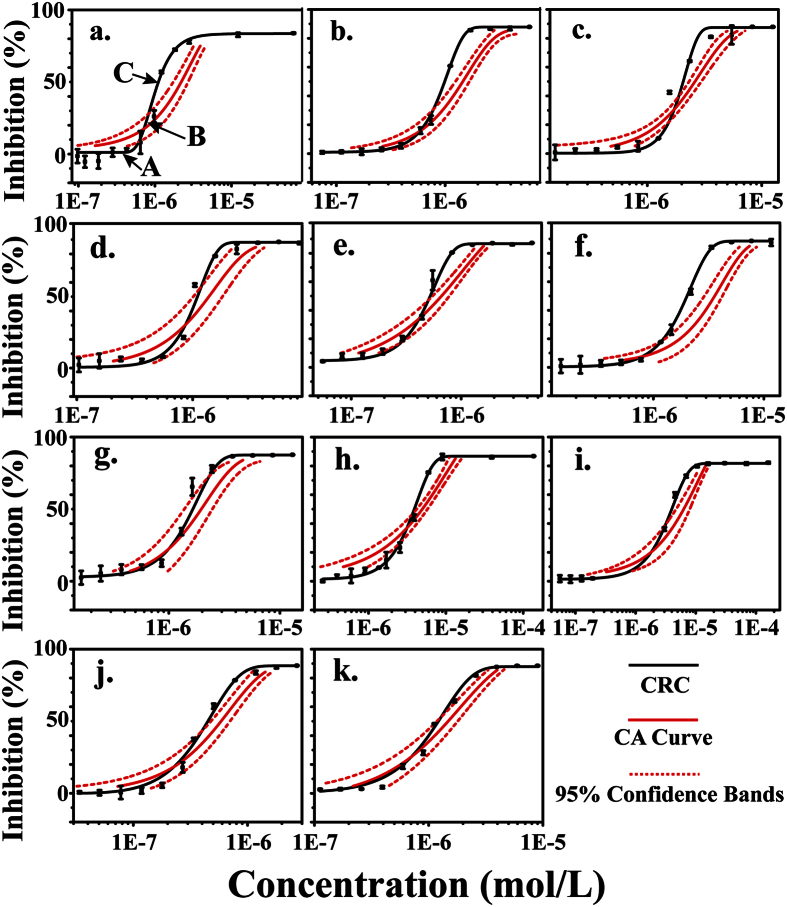
The CRCs for *E*. *coli* following exposure the binary mixture of SAs and ERY at 12 h show that they crossed the related CA curves with 95% confidence bands: (**a**) SCP and ERY; (**b**) SD and ERY; (**c**) SDX and ERY; (**d**) SM and ERY; (**e**) SMM and ERY; (**f**) SMP and ERY; (**g**) SMR and ERY; (**h**) SMZ and ERY; (**i**) SPY and ERY; (**j**) SQ and ERY; and (**k**) SSZ and ERY.

The CRCs for the mixture of SAs and ERY at 12 h are shown in Fig. 1. When the CA model was utilized to judge joint toxic actions, the CRCs for the mixtures crossed the CA curves, which expressed a heterogeneous pattern of joint toxic action. For example in Fig. 1(a), the CRC for the binary mixture of SCP and ERY was below the CA curves with 95% confidence bands in the low-concentration range (e.g. point A in the CRC), denoting an antagonistic effect; the CRC was located in the CA curves with 95% confidence bands in the medium-concentration range (e.g. point B in the CRC), denoting an additive effect; and the CRC was above the CA curves, with 95% confidence bands in the high-concentration range (e.g. point C in the CRC), denoting a synergistic effect.

We wondered whether the cross-phenomenon had a relationship with hormesis based on their similarity in the heterogeneous pattern of joint action and whether SAs or ERY can cause hormesis in *E*. *coli*.

### Hormesis based on the structural affinity of SAs with the SdiA protein

**Figure 2:**
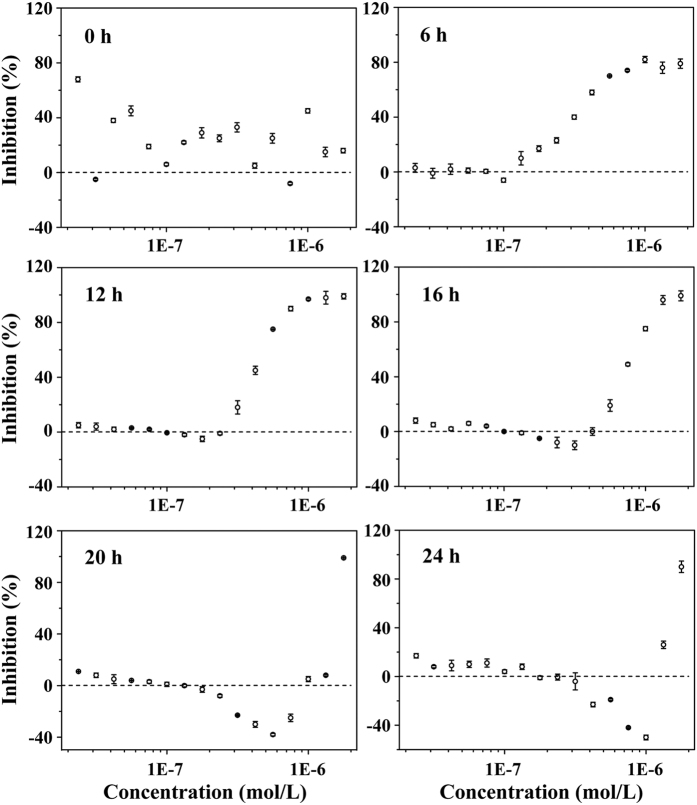
The toxicity of SCP to *E*. *coli* at 0, 6, 12, 16, 20 and 24 h in 0.4-fold LB culture medium.

To answer the questions proposed above, the growth curves for 24 hours of *E*. *coli* that were exposed to different concentrations of a mixture of SCP (as a representative SA) and ERY were determined in 0.4-fold LB culture medium to observe hormetic effects. The results shown in Figures S3 and S4 (briefly in Fig. 2) indicated that SCP could induce obvious hormesis in *E*. *coli*, whereas ERY had no hormetic effect on *E*. *coli*. The hormesis by SCP on *E*. *coli* was a time-dependent phenomenon which appeared at 12 h, with maximum stimulation reaching 40% at 24 h, and the maximum stimulation concentration increased from 1.33E-7 mol/L to 1.00E-6 mol/L. Then, why can SAs cause hormesis in *E*. *coli*?

#### Molecular docking between SAs and the SdiA protein

**Figure 3:**
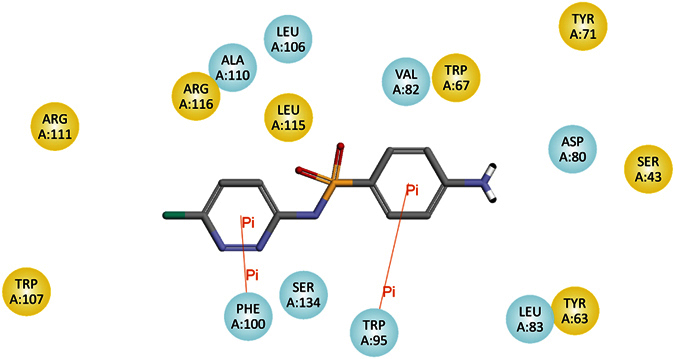
The interaction diagram showing the simulated interaction of SCP with the SdiA protein based on molecular docking studies.

Our previous study^18^ have demonstrated that the interaction between the LuxR protein and SAs could stimulate the expression of *luxR* mRNA, which led to hormesis of SAs in *Photobacterium phosphoreum*. According to molecular docking studies performed for SAs with the LuxR protein, interaction diagram of SCP with the SdiA protein (a LuxR homologue^25^) was simulated and is shown in Fig. 3 (other diagrams shown in Figure S5)^26^. It was found that there were at least two pi-cation bonds between SAs and the SdiA protein, which indicated that the protein could bind the ligand securely to the active site, and this binding mode was similar to that of the interaction between SAs and the LuxR protein^18^. Therefore, the structural affinity of SAs for the SdiA protein could be expressed as the specific binding between the SAs and the active site of the SdiA protein through at least two pi-cation bonds to form stable complexes. Could the interaction between SAs and SdiA protein stimulate the expression of *sdiA* mRNA?

#### The stimulation of the expression of sdiA mRNA by SAs

**Figure 4:**
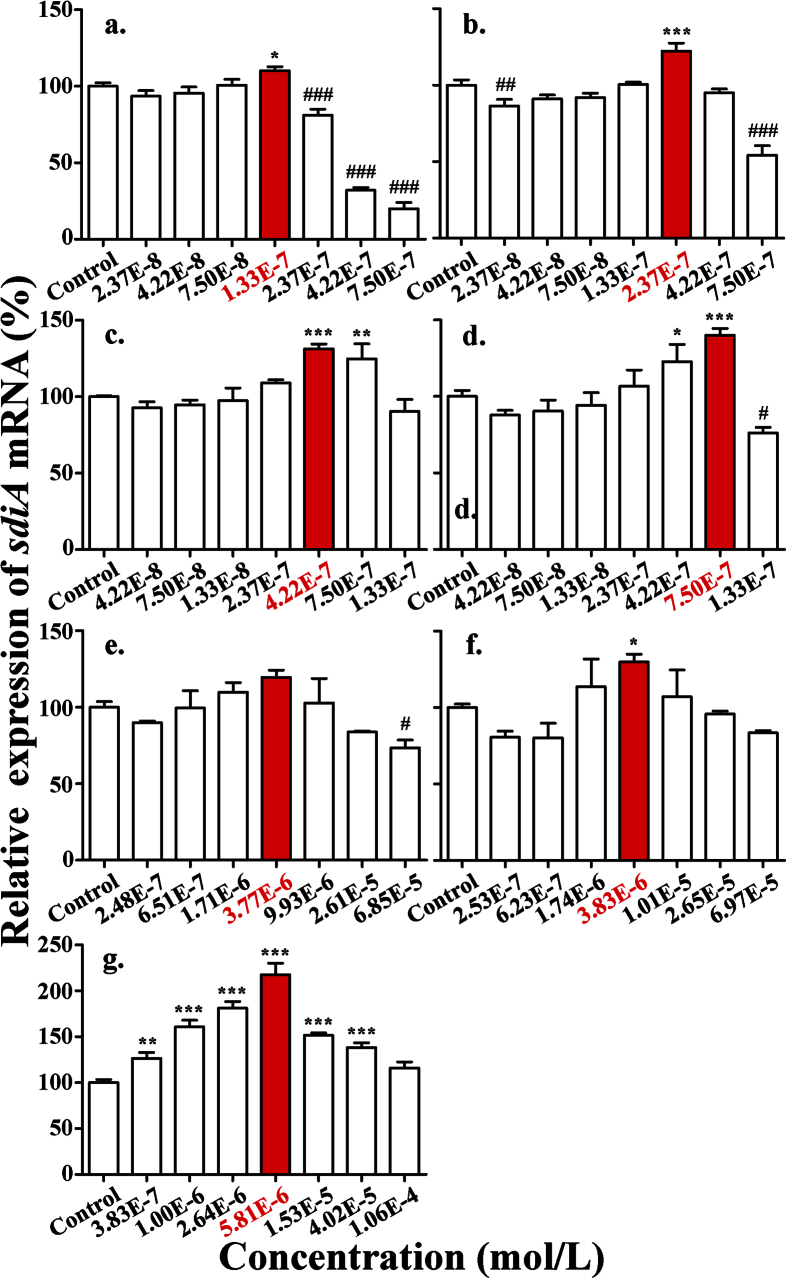
The expression of sdiA mRNA in *E*. *coli* exposed to various concentrations of SAs: (**a**) SCP at 12 h; (**b**) SCP at 16 h; (**c**) SCP at 20 h; (**d**) SCP at 24 h; (**e**) SMP at 24 h; (**f**) SMR at 24 h; and (**g**) SPY at 24 h. Differences were considered statistically significant at p < 0.05, p < 0.01 and p < 0.001, which are labeled with *, ** and *** for the increases in *sdiA* mRNA and ^#, ##^ and ^###^ for the decreases in *sdiA* mRNA, respectively.

The expression of *sdiA* mRNA in *E*. *coli* exposed to different concentrations of SCP (as a representative SA) was determined at 12 h (Fig. 4(a)), and showed that SCP at 1.33E-7 mol/L increased the expression of *sdiA* mRNA by 10% compared with the control. A stimulatory effect of SCP on the growth of *E*. *coli* was also observed at the same concentration point (Fig. 2). Previous studies have shown that the SdiA protein could promote or inhibit RNA polymerase binding to gene promoters, thereby affecting the transcription of target genes that plays an important role in the growth of *E*. *coli*^27^,^28^. Therefore, the excess SdiA protein induced by SCP might stimulate the growth of *E*. *coli*. In addition, the significant inhibition of SCP on the expression of *sdiA* mRNA at 2.37E-7, 4.22E-7 and 7.50E-7 mol/L was in accord with an obvious inhibitory effect of SCP on the growth of *E*. *coli*. These results indicated that the effect of SCP on the expression of *sdiA* mRNA coincided with the hormetic effects by SCP on *E*. *coli*. Because hormesis by SCP on *E*. *coli* was a time-dependent process, the expression of *sdiA* mRNA was also determined at 16, 20 and 24 h to observe whether it was time-dependent. As shown in Fig. 4(b–d), with increasing exposure time, the stimulatory effect of SCP on *sdiA* mRNA expression increased gradually from 10% to 40%, and the stimulatory concentration range shifted from 1.33E-7 mol/L to 7.50E-7 mol/L, which showed a high relevance with regard to the time-dependence of hormesis. In summary, the hormetic effects of SAs on *E*. *coli* was induced by the additional expression of *sdiA* mRNA resulting from the interaction between SAs and the SdiA protein.

### The time-dependent cross-phenomenon

**Figure 5:**
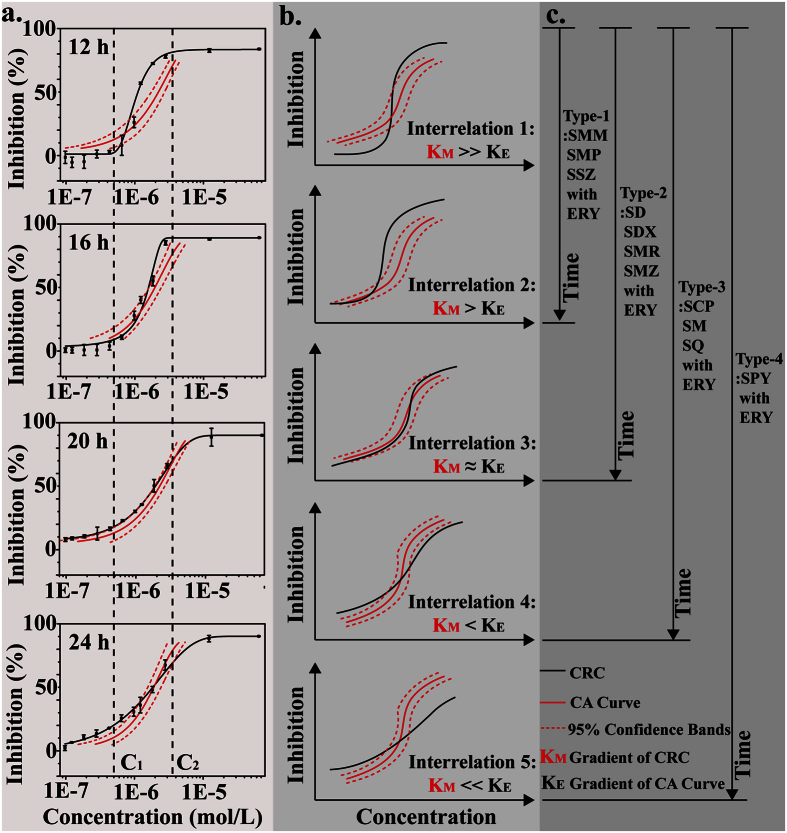
(**a**) The CRCs for *E*. *coli* following exposure to the binary mixture of SCP and ERY at 12, 16, 20 and 24 h with the related CA curves; (**b**) the five types of interrelations between the CRCs for mixtures and the CA curves; (**c**) the four types of mixtures divided into groups based on the different variation tendencies from Interrelation-1 to other types of interrelations with increasing exposure time.

Considering that the hormesis by SCP on *E*. *coli* was proven to be a time-dependent phenomenon, it was reasonable for us to suspect that this time-dependent hormesis could induce a time-dependent cross-phenomenon. Figure 5a shows the corresponding CRCs for *E*. *coli* exposed to a binary mixture of SCP and ERY at 12, 16, 20 and 24 h (other CRCs are plotted in Figure S6). It could be observed that the cross-phenomenon varied with increasing exposure time, and different SAs had different variation tendencies. To investigate this time-dependent cross-phenomenon, the interrelation between the gradient of the CRC and CA curve was expressed by the K_E_ and K_M_, respectively; moreover, K_E_ is an important indicator of the CRC and is always applied to represent the characterizations of the CRC^29^,^30^,^31^. There are five types of interrelations between the K_E_ and K_M_ of binary mixtures (Fig. 5b): Interrelation-1 (K_M_ ≫ K_E_), Interrelation-2 (K_M_ > K_E_), Interrelation-3 (K_M_ ≈ K_E_), Interrelation-4 (K_M_ < K_E_), and Interrelation-5 (K_M_ ≪ K_E_).

For all mixtures, the interrelations between K_E_ and K_M_ shifted from Interrelation-1 at 12 h toward other interrelation types with increasing exposure time, expressed as different variations for different mixtures. Accordingly, the binary mixtures of SMM, SMP and SSZ with ERY had a similar variation tendency, classified as Type-1 (from Interrelation-1 to Interrelation-2); the binary mixtures of SD, SDX, SMR and SMZ with ERY had a similar variation tendency, classified as Type-2 (from Interrelation-1 to Interrelation-3); the binary mixtures of SCP, SM and SQ with ERY had a similar variation tendency, classified as Type-3 (from Interrelation-1 to Interrelation-4); and the binary mixture of SPY with ERY was classified as Type-4 (from Interrelation-1 to Interrelation-5).

In summary, binary mixtures of SAs and ERY have been divided into four types based on the different variations of interrelations between the K_E_ and K_M_ and are expressed as different time-dependent cross phenomena. As demonstrated above, the stimulatory effects of SAs on the expression of *sdiA* mRNA were closely related with time-dependent hormesis; thus, we desired to know whether the different stimulation of SAs on *sdiA* mRNA took effect and this issue is discussed below.

## Discussion

**Figure 6:**
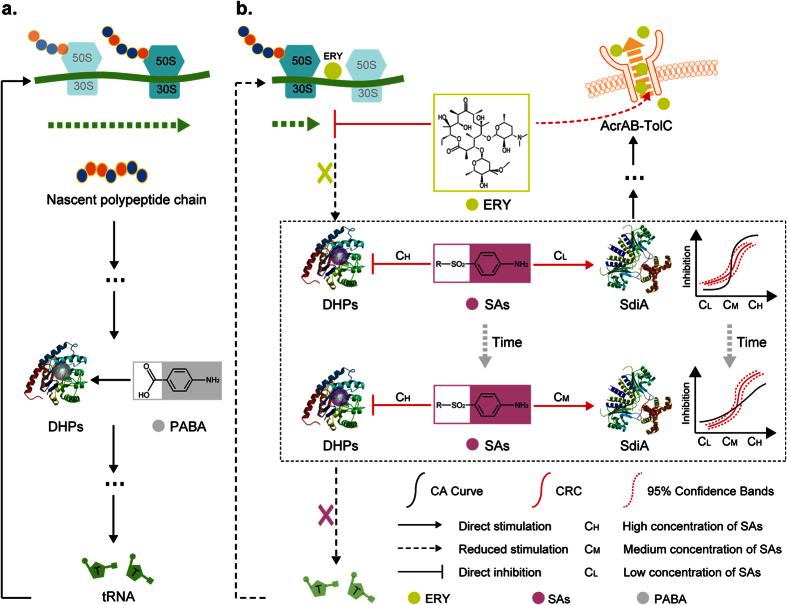
(**a**) The loop circuit in *E*. *coli* that relates to the time-dependent cross-phenomenon; (**b**) the mechanistic hypothesis for the time-dependent cross-phenomenon in the presence of SAs and ERY.

As a macrolide antibiotic, ERY can bind to its target site, the 50S ribosomal subunit, through irreversible binding, interdicting transpeptidation and mRNA displacement and thus selectively inhibiting protein synthesis^32^. SAs could compete with para-aminobenzoic acid to bind dihydropteroate synthase (Dhps), impeding the synthesis of dihydrofolic acid, which could be utilized to synthesize ribotides to form ribosomes^33^. Hence, SAs and ERY could produce a double block in the loop circuit of bacteria when applied together, exhibiting a synergistic effect. In addition, low concentrations of SAs could stimulate the growth of bacteria by binding to the SdiA protein and promoting the expression of *sdiA* mRNA. The overexpression of the SdiA protein would affect the expression of a battery of genes, including multidrug efflux pump genes^34^. We hypothesized that excess SdiA protein would increase the expression of the AcrAB-TolC efflux pump, which has been proven to have the ability to discharge ERY from *E*. *coli*^35^. Thus, mixtures of SAs and ERY at low concentrations exhibited an antagonistic effect on the growth of *E*. *coli*. In conclusion, the hormetic effect of SAs on *E*. *coli* resulted in the cross-phenomenon at 12 h, as shown in Fig. 6.

It was proven that the cross-phenomenon for SAs and ERY varied with increasing exposure time, and we wanted to determine how time-dependent hormesis of SAs on *E*. *coli* caused this time-dependent cross-phenomenon. For the mixture of SCP and ERY (Fig. 5a, C_1_ and C_2_ set as representative values for the low concentration and high concentration, respectively), as an example, the antagonistic effect of the mixture at a low concentration (C_1_) switched to synergistic effect because the effect of SCP on the expression of *sdiA* mRNA changed from stimulation to inhibition with increasing exposure time (Fig. 4(a–d)); the stimulatory concentration range of SCP on *sdiA* mRNA increased gradually to a high-concentration, and thus the joint toxic action of the mixture at the C_2_ concentration changed from synergistic to antagonistic. To summarize, the joint toxic actions of the mixture at a specific concentration range varied with increasing exposure time. The mechanistic hypothesis for the time-dependent cross-phenomenon described above is depicted in Fig. 6. These results indicated that the increased expression of *sdiA* mRNA led to the time-dependent hormesis of *E*. *coli* in response to SAs, which was a key factor involved in the time-dependent cross-phenomenon.

The time-dependent cross phenomena of binary mixtures showed different variation tendencies with increasing exposure time, which might have been caused by the different stimulatory effects of SAs on the expression of *sdiA* mRNA. SMP, SMR, SCP and SPY were selected as representatives of the four types of SAs, and the expression of *sdiA* mRNA in *E*. *coli* exposed to these four chemicals was determined at 24 h, as shown in Fig. 4(d–g). It was observed that the order of maximal stimulation was SMP (20% of the control, Type-1) < SMR (30% of the control, Type-2) < SCP (40% of the control, Type-3) < SPY (118% of the control, Type-4), and the Type-1 and Type-2 stimulatory effects of SAs were relatively lower than those of Type-3 and Type-4. Although the interrelations between the CRCs and the CA curves with 95% bands all varied with increasing exposure time (see Figure S6(e,f,h) and Fig. 5(a)), the changes in joint toxic actions of SAs and ERY in Type-1 and Type-2 were not as obvious as those in Type-3 and Type-4. Figure S6(e,f) shows the detailed CRCs of binary mixtures of SMP and SMR with ERY and the relative CA curves at 12, 16, 20 and 24 h. With increasing exposure time, the antagonistic effect of the mixture at low concentrations shifted to an additive or synergistic effect because the stimulation concentration range of the SAs increased to a high concentration; the synergism at the high concentration remained steady or changed to addition because the stimulatory effects level of SAs on the expression of *sdiA* mRNA were limited in these two types of interrelations. In conclusion, when the concentrations of the mixtures were low, the stimulatory effects of SAs on *sdiA* mRNA had a significant impact on the expression of the AcrAB-TolC efflux pump, resulting in the discharge of low concentration ERY at 12 h. With increasing exposure time, the stimulatory concentration of SAs increased gradually into the high-concentration range, in which the ERY concentration was high. In this instance, if the stimulation provided by SAs was limited, with a limited discharge of ERY, the joint toxic actions of SAs and ERY would remain synergistic or would change to additive; if the stimulatory effect of SAs was high enough to discharge a large portion of ERY, the joint toxic action of the agent would switch to antagonism. Therefore, the different stimulatory effects of SAs on the expression of *sdiA* mRNA led to four different types of binary mixtures of SAs and ERY, which presented different variation tendencies for the time-dependent cross-phenomenon.

## Materials and Methods

### Chemicals and organism

Eleven SAs and ERY were all purchased from Sigma (St Louis, MO) without further purification (purity ≥ 99%), and the details are listed in Table 1. Freeze-dried *E*. *coli* (MG1655) were obtained from Biovector Science Lab, Inc. The Luria-Bertani (LB) culture medium consisted of NaCl, yeast extract, tryptone, and distilled water, and the pH was adjusted to 7.0 ± 0.2. Before each test, the bacteria were inoculated from a stock culture that was maintained on culture medium agar at 4 °C, transferred to a fresh agar plate and cultured at 37 ± 1 °C for 12 hours. The bacteria were further grown in a liquid culture medium by shaking (160 rev/min) at 37 ± 1 °C for 6 hours until the final bacteria density reached approximately 1,000 cells/mL for the toxicity tests.

### Toxicity tests

The chemicals were dissolved in 0.1% DMSO and then diluted with 1% NaCl solution to appropriate concentrations for the toxicity tests. A series of chemical and bacterial solutions were successively added into the 96-well plates, which were cultured at 37 ± 1 °C. The initial and final optical density (OD) values were determined at 600 nm on a Bioscreen C MBC automatic growth curve analyzer (Bioscreen, Finland). The toxicity results are expressed through the inhibition (Y) of the OD value:


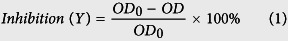


where OD_0_ is the average OD value of *E*. *coli* in the absence of chemicals, and OD is the average OD value following when exposure to the test chemicals (triplicate experiments). When the EC_50_ of each chemical was obtained, a series of binary mixtures of SAs and ERY were designed based on equitoxic ratios using these EC_50_ values^36^. The mixture toxicity tests were performed using the same method applied in the single toxicity tests. Finally, the observed concentration-response data were fitted by a Weibull regression model.

### CA model

CA is a prominent reference model used for the evaluation of joint effects. For a multi-component mixture of n substances, CA can be defined by^37^:


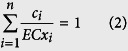


where c_i_ is the concentration of component i when the total effect of the mixture is x%; ECx_i_ is the concentration of compound i at which i applied individually provokes the same effect (x%) as the mixture. c_i_ can also be expressed as the relative proportion, p_i_, of the total concentration, c_mix_, and p_i_ = c_i_/c_mix_. Eq. (3) can be rearranged to the following mathematical formula^37^:


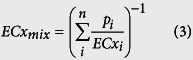


where ECx_mix_ is the mixture concentration provoking x% toxicity effects. The CA curves were also fitted by a Weibull regression model with the statistical uncertainties expressed as 95% confidence bands. Because the CA model is applied based on the assumption that the individual mixture components have similar modes of action, the comparison between the CA curve and the actual CRC for the mixture can be used to determine the joint toxic action: when the CRC is below the CA curves with 95% confidence bands, the joint toxic action is antagonism; when the CRC is located in the CA curves with 95% confidence bands, the joint toxic action is addition; when the CRC is above the CA curves with 95% confidence bands, the joint toxic action is synergism^12^.

### Molecular docking study

Molecular docking was performed with Discovery Studio 3.1 (DS, Accelrys Software, San Diego, CA, USA) using the CDOCKER protocol with the default parameters. The three-dimensional structures of the chemicals (ligands) used in the molecular docking were generated by Chemoffice, and the ligands were energy-minimized using the CHARMm force field before performing the docking. The protein crystal structure of SdiA (4LGW) in *E*. *coli* was obtained from the Protein Data Bank (http://www.pdb.org). Based on CDOCKER, the substrate orientation that gave the lowest interaction energy was chosen for post-docking analysis, and the binding situation that resulted in the lowest docking energy was used for further analyses^18^,^38^.

### Determination of the expression of *sdiA* mRNA

The expression of *sdiA* mRNA was determined by detecting the corresponding expression of mRNA^26^,^28^. The total RNA from *E*. *coli* was purified with Trizol, and 1 μg of total RNA was used for reverse transcription with random primers (Invitrogen) and SuperScript III (Invitrogen). Quantitative polymerase chain reaction was performed on a CFX Connect Real-Time PCR System (Bio-Rad) with SYBR green detection PCR Mastermix (Bio-Rad). All reactions were performed in duplicate with the following PCR parameters: 95 °C for 5 min followed by 40 cycles of 95 °C for 30 s, 55 °C for 30 s and 72 °C for 30 s, with the primer sequences listed in Table S1. These results are presented as the mean ± standard deviation. After the Kolmogorov-Smirnov test, a one-way analysis of variance (ANOVA) was used to determine the differences among treated and control groups, followed by Tukey’s multiple comparison test. Differences were considered statistically significant at p < 0.05, p < 0.01 and p < 0.001, which are labeled with *, ** and *** for an increase in *sdiA* mRNA expression and ^#, ##^ and ^###^ for a decrease in *sdiA* mRNA expression, respectively.

## Additional Information

**How to cite this article**: Sun, H. *et al*. Mechanism Underlying Time-dependent Cross-phenomenon between Concentration-response Curves and Concentration Addition Curves: A Case Study of Sulfonamides-Erythromycin mixtures on *Escherichia coli*. *Sci. Rep.* **6**, 33718; doi: 10.1038/srep33718 (2016).

## Supplementary Material

Supplementary Information

## Figures and Tables

**Table 1:** The details of the tested drugs.

Chemical name	Abbreviation	CAS	Relative molecular weight (g/mol)	EC_50_ (mol/L, 12 h)
Sulfachloropyridazine	SCP	80-32-0	284.72	(5.08 ± 0.44) × 10^−6^
Sulfadiazine	SD	68-35-9	250.28	(5.00 ± 0.49) × 10^−6^
Sulfadoxine	SDX	2447-57-6	310.33	(8.55 ± 0.12) × 10^−6^
Sulfameter	SM	651-06-9	280.30	(4.38 ± 0.44) × 10^−6^
Sulfamonomethoxine	SMM	1220-83-3	280.30	(1.56 ± 0.12) × 10^−6^
Sulfamethoxypyridazine	SMP	80-35-3	280.30	(4.93 ± 0.21) × 10^−5^
Sulfamerazine	SMR	127-79-7	264.30	(3.72 ± 1.23) × 10^−6^
Sulfamethazine	SMZ	57-68-1	278.33	(1.94 ± 0.13) × 10^−5^
Sulfapyridine	SPY	144-83-2	249.29	(4.58 ± 0.13) × 10^−5^
Sulfaquinoxaline	SQ	59-40-5	300.34	(1.94 ± 0.03) × 10^−6^
Sulfisoxazole	SSZ	127-69-5	267.30	(4.40 ± 0.52) × 10^−6^
Erythromycin	ERY	114-07-8	733.93	(1.70 ± 0.26) × 10^−7^
