# Corrigendum: Mechanism Underlying Time-dependent Cross-phenomenon between Concentration-response Curves and Concentration Addition Curves: A Case Study of Sulfonamides-Erythromycin mixtures on *Escherichia coli*

**DOI:** 10.1038/srep35899

**Published:** 2016-12-07

**Authors:** Haoyu Sun, Hongming Ge, Min Zheng, Zhifen Lin, Ying Liu

Scientific Reports 6: Article number: 3371810.1038/srep33718; published online: 09 20 2016; updated: 12 07 2016

This Article contains an error in the Acknowledgements section:

“This work was funded by the Foundation of the State Key Laboratory of Pollution Control and Resource Reuse, China (PCRRY11003), the National Natural Science Foundation of China (21377096, 21577105), the “Climbing” Program of Tongji University (0400219287), the 111 Project, and the Science & Technology Commission of Shanghai Municipality (14DZ2261100)”.

should read:

“This work was funded by the Foundation of the State Key Laboratory of Pollution Control and Resource Reuse, China (PCRRK16007), the National Natural Science Foundation of China (21377096, 21577105), the “Climbing” Program of Tongji University (0400219287), the 111 Project, and the Science & Technology Commission of Shanghai Municipality (14DZ2261100)”.

