# Supplementary material for: Mechanism Underlying Time-dependent Cross-phenomenon between Concentration-response Curves and Concentration Addition Curves: A Case Study of Sulfonamides-Erythromycin mixtures on Escherichia coli
Source: Sci Rep. 2016 Sep 20;6:33718. doi: 10.1038/srep33718 (PMC5028747; doi:10.1038/srep33718)
Supplement: Supplementary Information [file srep33718-s1.pdf]

Supplementary Information

Mechanism Underlying Time-dependent Cross-Phenomenon between Concentration-response Curves and Concentration Addition Curves: A Case Study of Sulfonamides-Erythromycin mixtures on *Escherichia coli*

Haoyu Sun [a, 1], Hongming Ge [a, 1], Min Zheng [a], Zhifen Lin [a, b, *], Ying Liu [c]

[a] *State Key Laboratory of Pollution Control and Resource Reuse, College of Environmental Science and Engineering, Tongji University, Shanghai 200092, China*

[b] *Collaborative Innovation Center for Regional Environmental Quality, China*

[c] *Shanghai Key Laboratory of Chemical Assessment and Sustainability, Shanghai, China*

[1] These two authors contributed equally to this work and are co-first authors.

Corresponding Author

[*]Zhifen Lin. Email: lzhifen@tongji.edu.cn. Tel.: +86-021-65983336. Fax: +86-021-65982688. Address: College of Environmental Science and Engineering, Tongji University, 1239 Siping Road, Shanghai 200092, China.

Number of pages: 14

Number of figures: 6

Number of tables: 1

Figure S1. The superiority of CA model than TU……………………………………S3

Figure S2. CRCs for individual toxicities of ERY and SAs to *E. coli*………………S4

Figure S3. Growth curves of *E.coli* in SCP and ERY………………………………S6

Figure S4. Toxicity of SCP to *E. coli* for 24 hours…………………………………S7

Figure S5. Interaction diagrams of SAs with SdiA protein…………………………S9

Figure S6. CRCs for combined toxicities of SAs and ERY to *E. coli*……………S10

Table S1. Primer sequences of *rrsG* and *sdiA*……………………………………S14

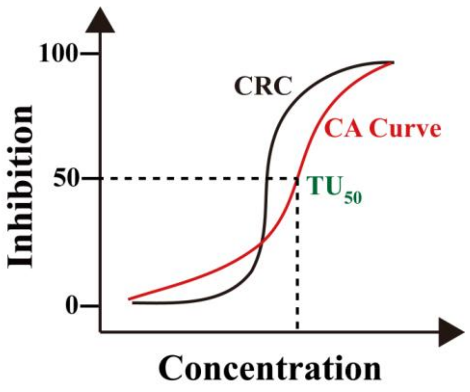

Figure S1. The superiority of CA model, compared with the toxic indexes (TU_{50} index as a representative), can be expressed in this figure when the joint toxic action of several mixtures varies with dose on a given testing organism, which is that CA can more comprehensively reflect the joint toxic action than TU_{50} at the whole concentration range. TU_{50} value can be calculated by the following equation: $TU_{50} = \frac{C_A}{EC_{50A}} + \frac{C_B}{EC_{50B}}$, where C_A and C_B are the concentrations of chemicals A and B in the binary mixture at the median inhibition; EC_{50A} and EC_{50B} represent the median effective concentrations of the single chemicals A and B, respectively. CA model can be defined by: $\sum_{i=1}^{n} \frac{c_i}{ECx_i} = 1$, where c_i is the concentration of component i when the total effect of the mixture is x%; ECx_i is the concentration of compound i at which i applied individually provokes the same effect (x%) as the mixture. Therefore, TU_{50} approach just determines the joint toxic action (synergism) of combined chemicals at a concentration point, however, there is a heterogeneous pattern of joint toxic action when CA model is used to predict or judge the joint toxic action. For the mathematical formulation of the CA model, quotient c_i/ECx_i expresses the concentration of mixture components as fractions of equi-effective individual concentrations, which is a dimensionless concept that has been termed a toxic unit (TU).

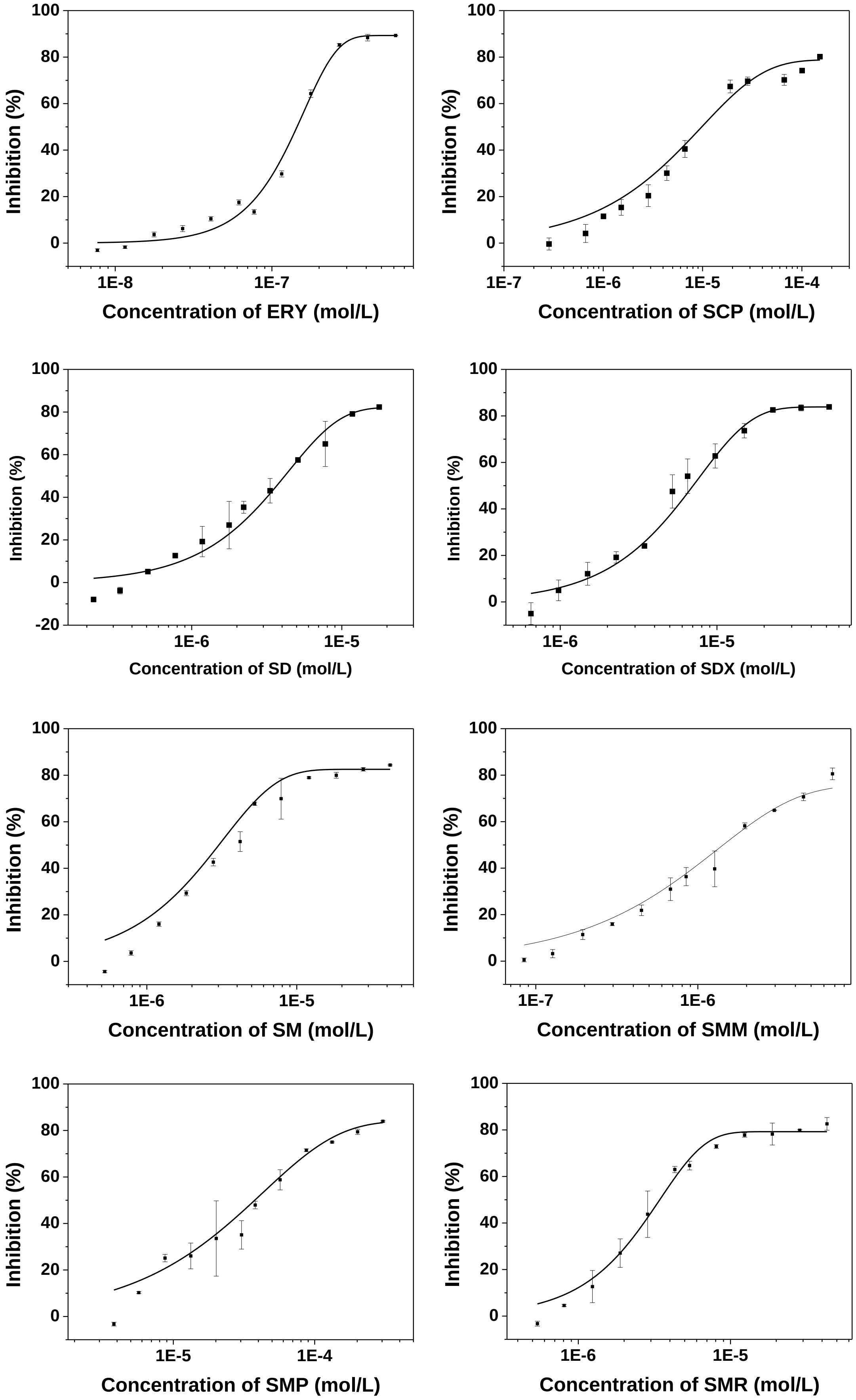
Inhibition (%)
Concentration of ERY (mol/L)
Concentration of SCP (mol/L)
Concentration of SD (mol/L)
Concentration of SDX (mol/L)
Concentration of SM (mol/L)
Concentration of SMM (mol/L)
Concentration of SMP (mol/L)
Concentration of SMR (mol/L)

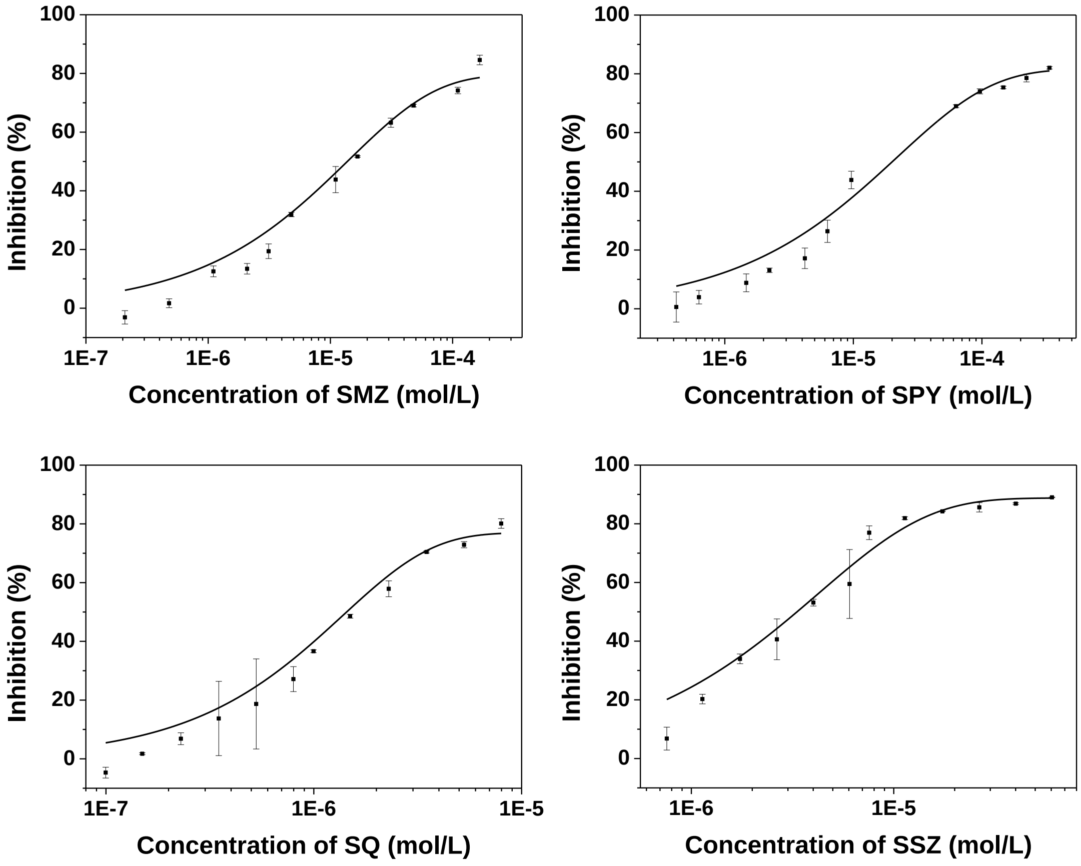

Figure S2. The CRCs for individual toxicities of ERY and SAs to *E.coli* at 12 h.

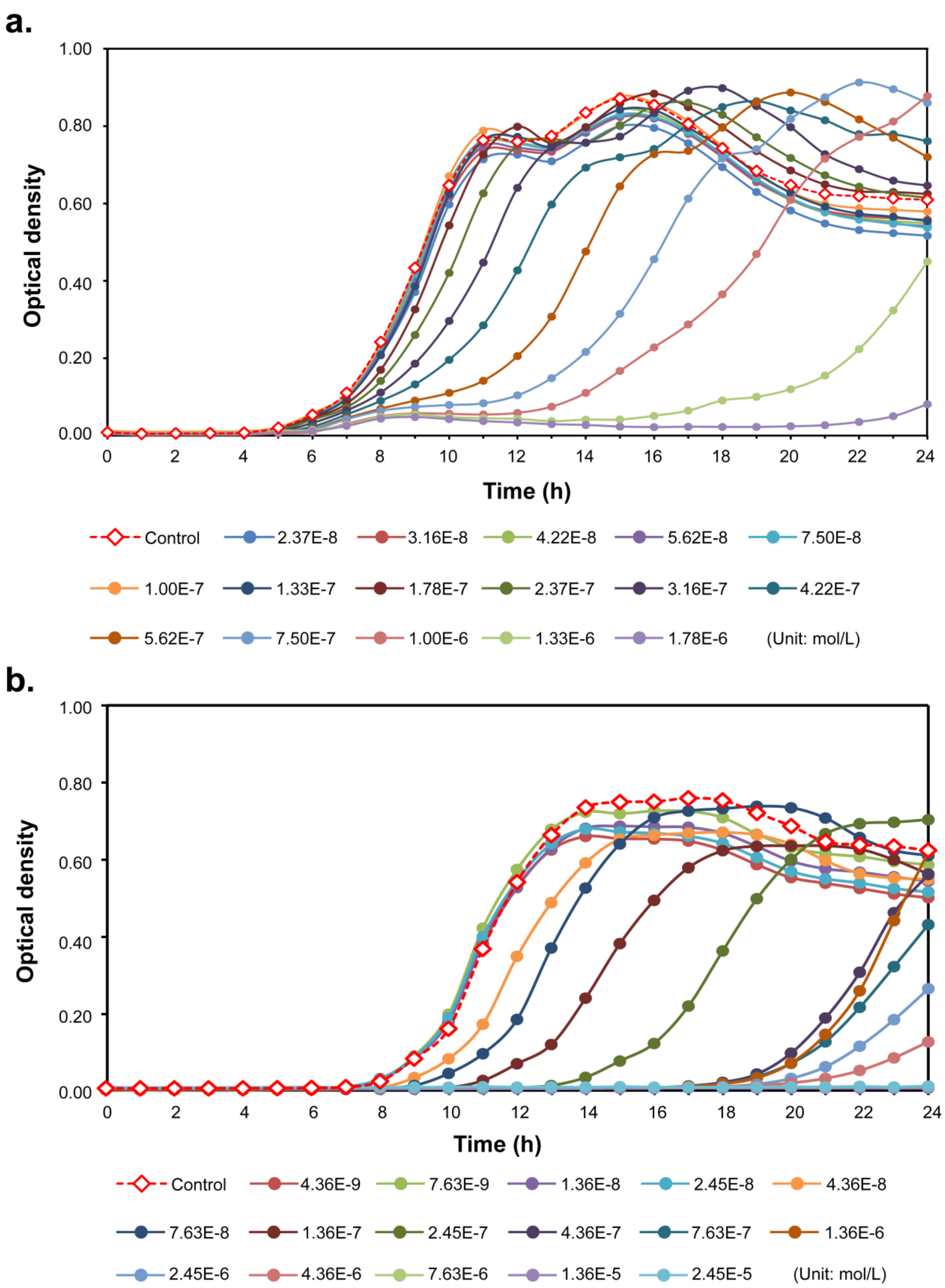

Figure S3. The growth curves of *E.coli* exposed to different concentrations of SCP (a) and ERY (b) for 24 hours in 0.4-fold LB culture medium.

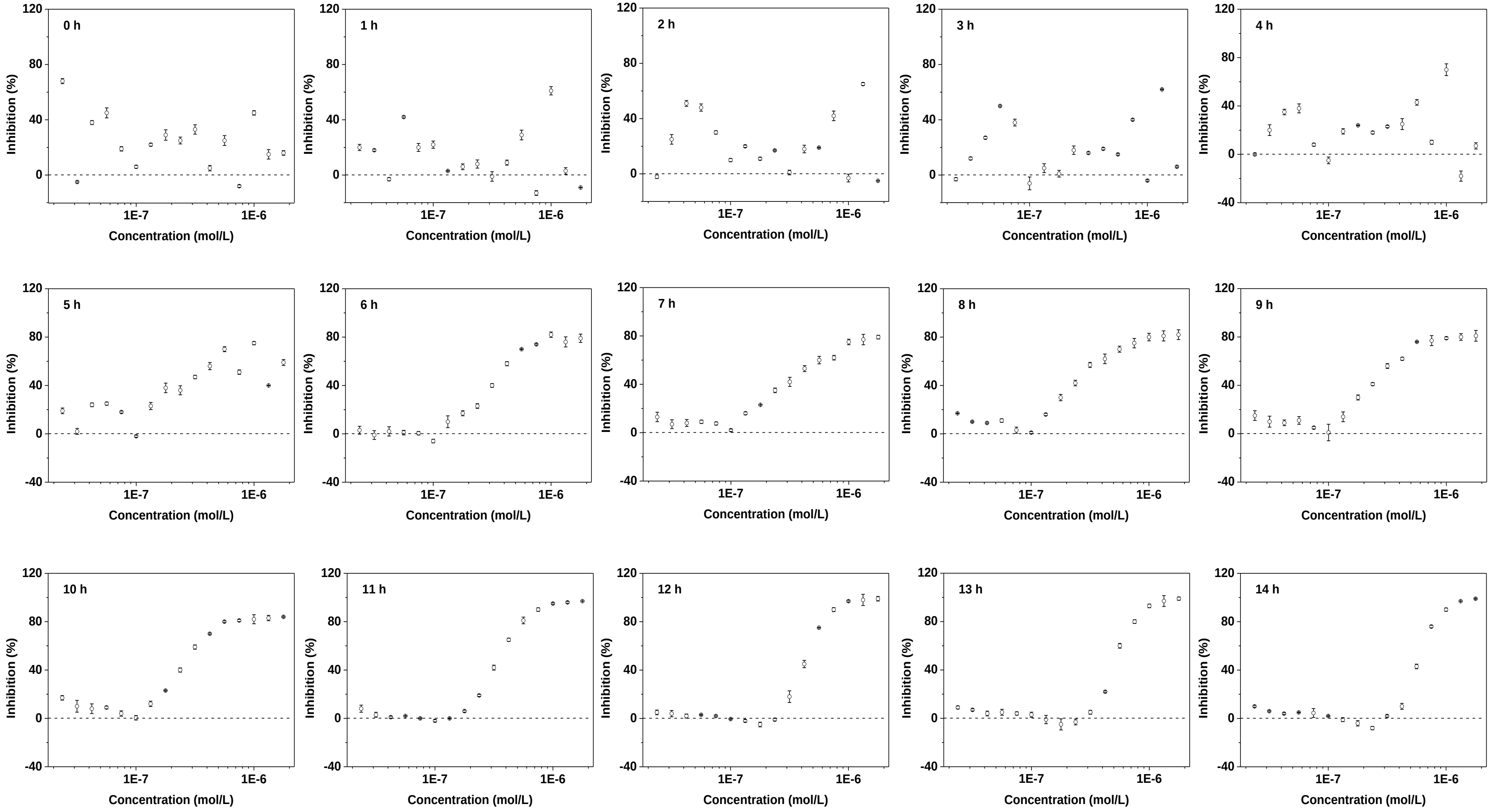
0 h
1 h
2 h
3 h
4 h
5 h
6 h
7 h
8 h
9 h
10 h
11 h
12 h
13 h
14 h
Inhibition (%)
Concentration (mol/L)
120
80
40
0
-40
1E-7
1E-6

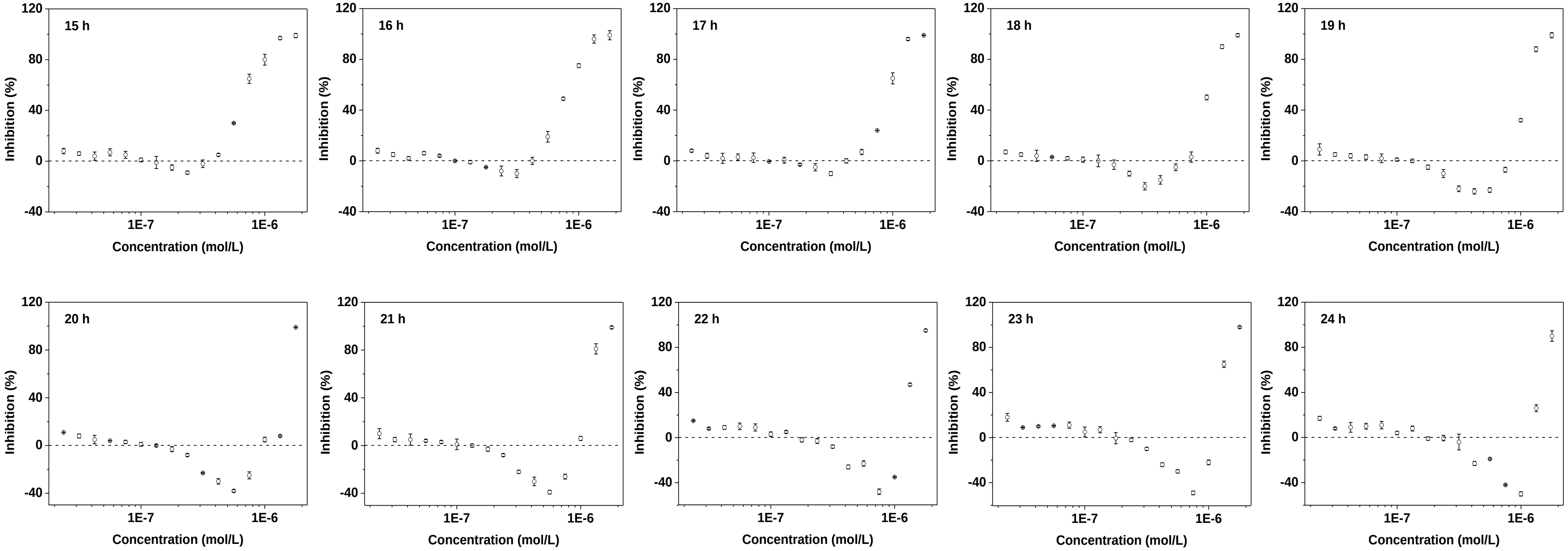

Figure S4. The toxicity of SCP to *E.coli* for 24 hours in 0.4-fold LB culture medium.

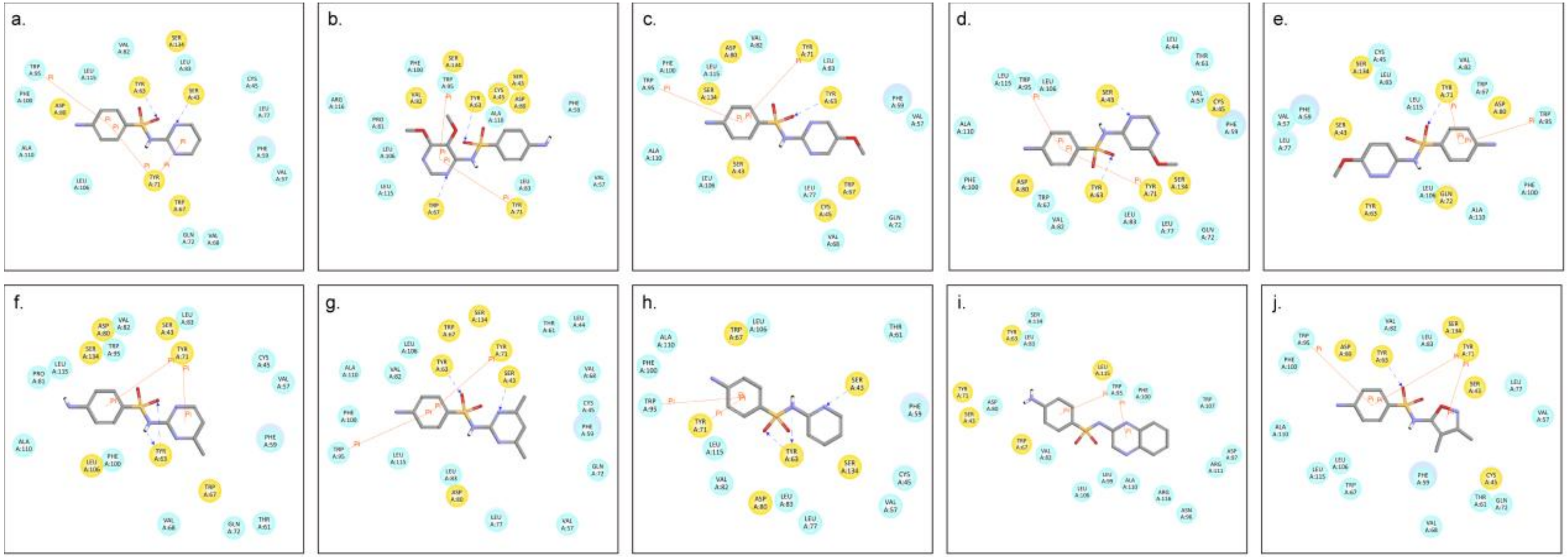

Figure S5. The interaction diagrams showing the simulated interactions of SAs with the SdiA protein simulated based on molecular docking studies: (a) SD; (b) SDX; (c) SM; (d) SMM; (e) SMP; (f) SMR; (g) SMZ; (h) SPY; (i) SQ; and (j) SSZ.

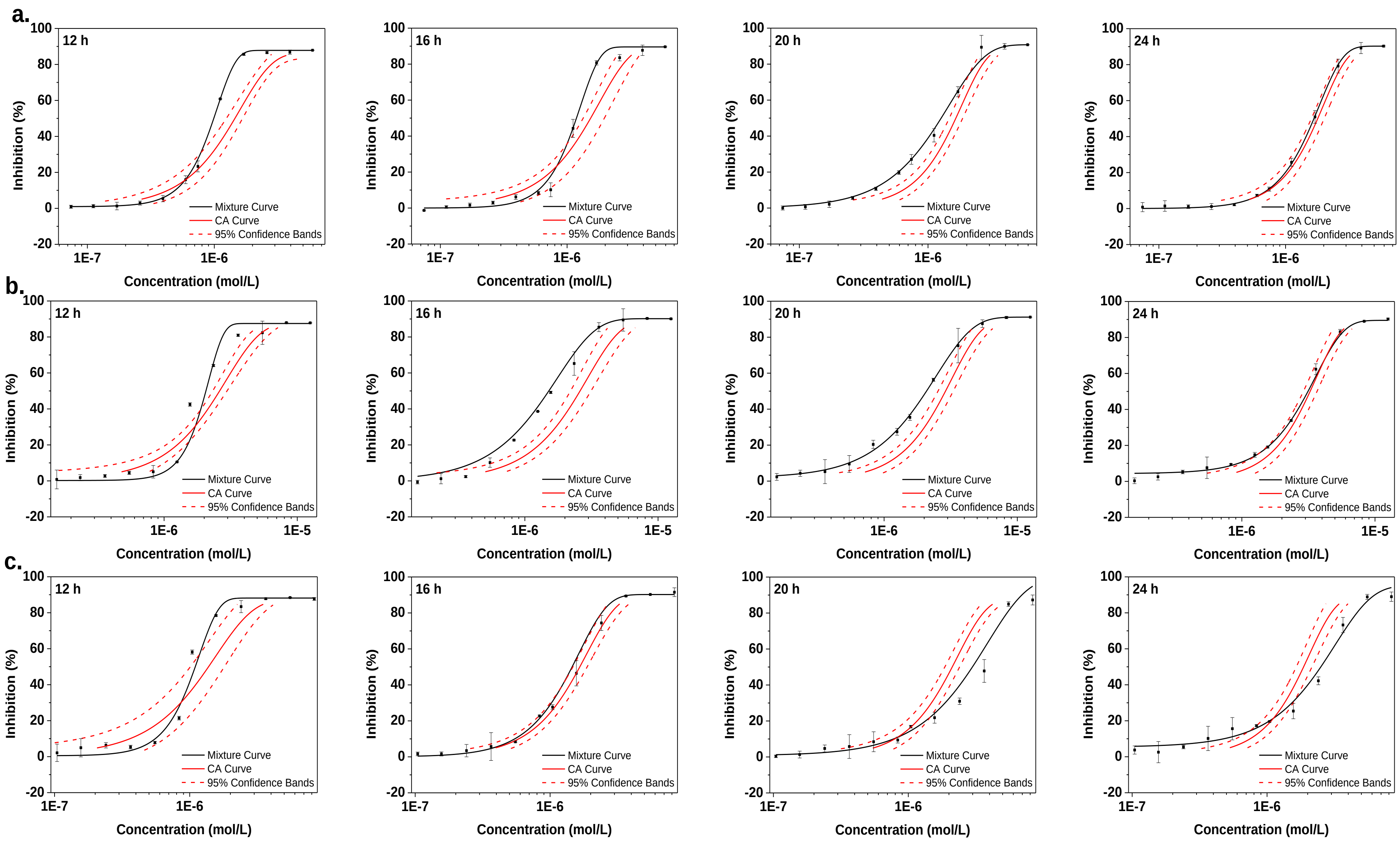
a.
b.
c.
12 h
16 h
20 h
24 h
Inhibition (%)
Concentration (mol/L)
Mixture Curve
CA Curve
95% Confidence Bands
1E-7
1E-6
1E-5
100
80
60
40
20
0
-20

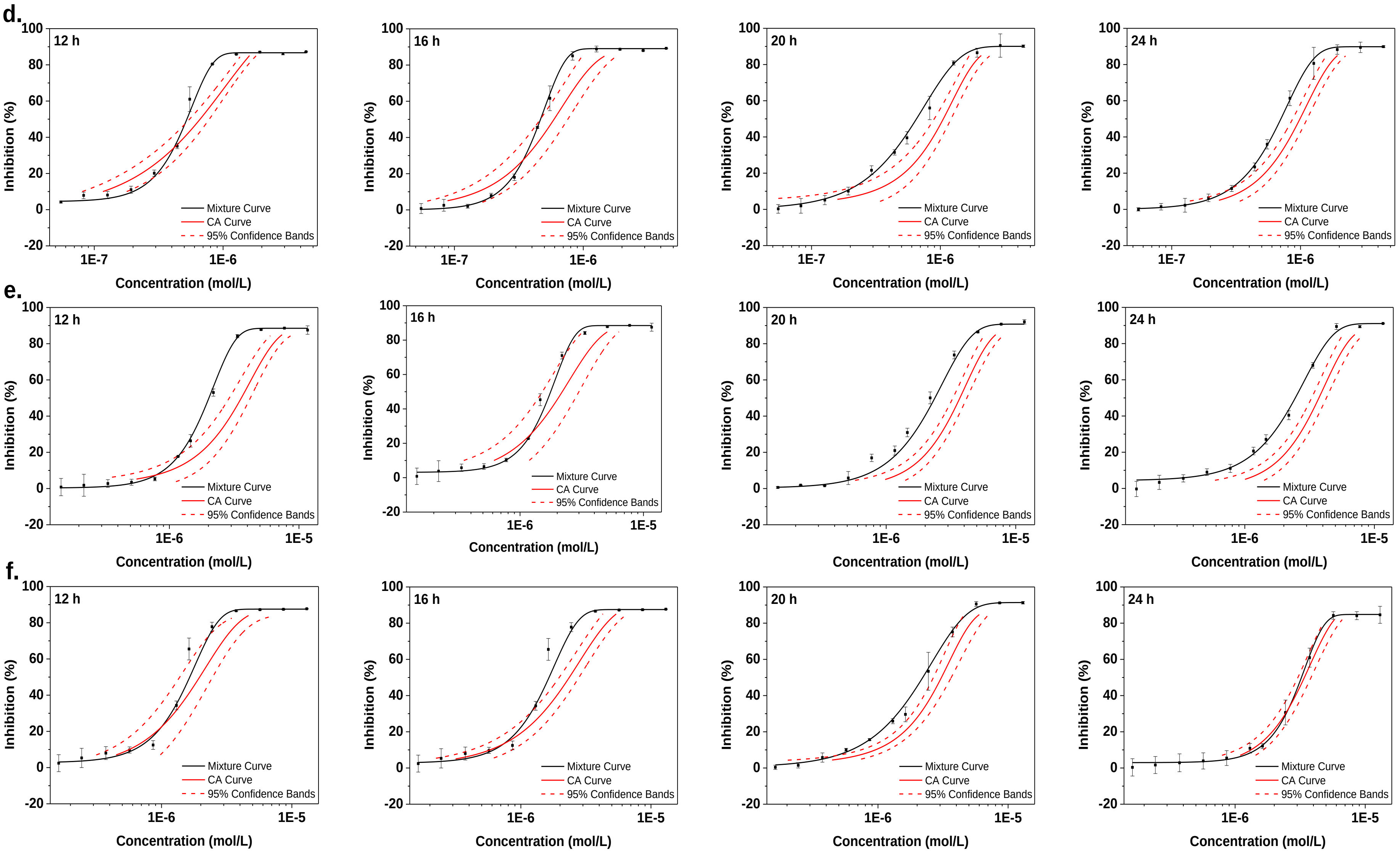

d.
12 h
16 h
20 h
24 h
e.
12 h
16 h
20 h
24 h
f.
12 h
16 h
20 h
24 h
Inhibition (%)
Concentration (mol/L)
1E-7
1E-6
1E-5
Mixture Curve
CA Curve
95% Confidence Bands

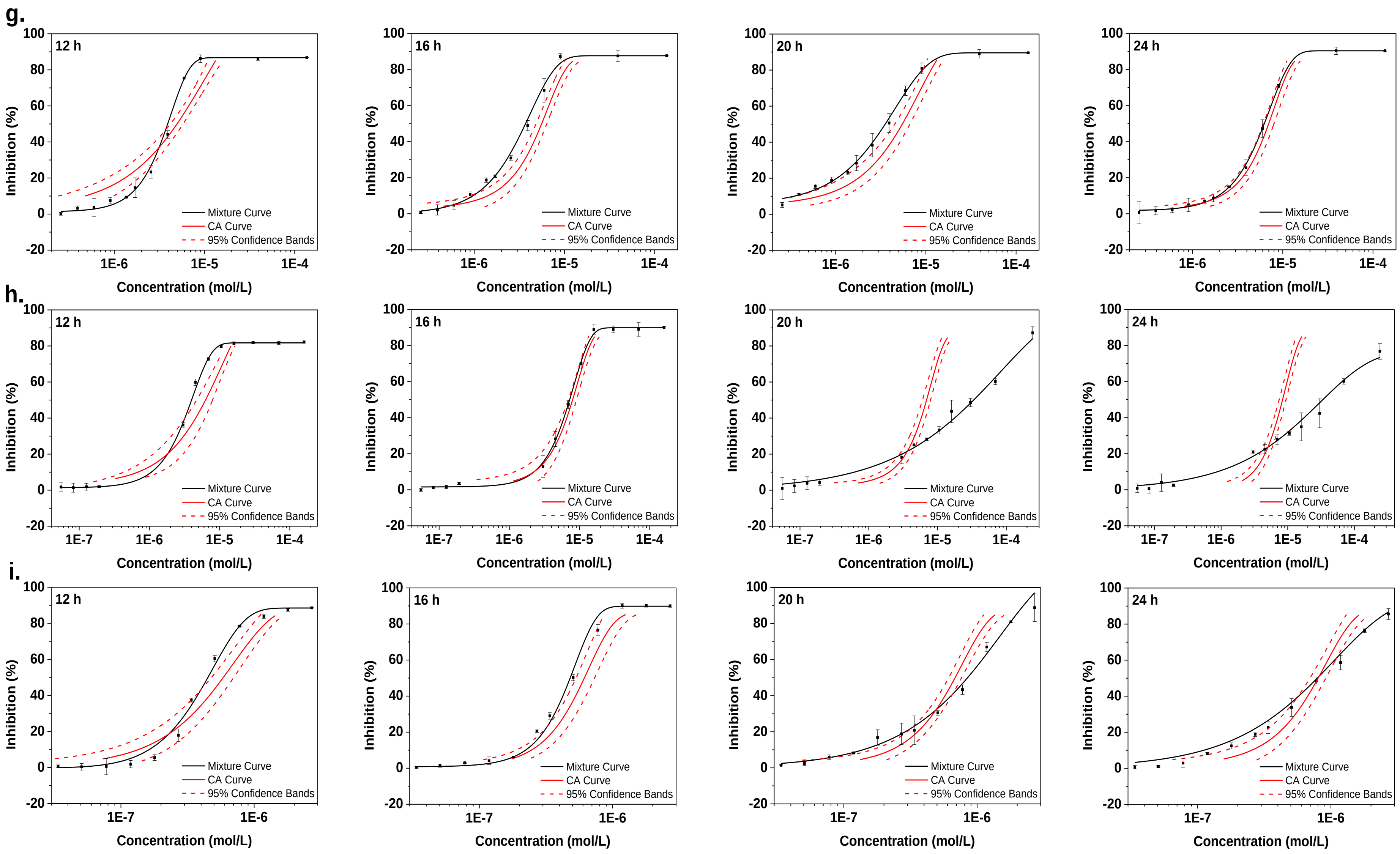
g.
h.
i.
12 h
16 h
20 h
24 h
Inhibition (%)
Concentration (mol/L)
Mixture Curve
CA Curve
95% Confidence Bands
1E-7
1E-6
1E-5
1E-4

j.

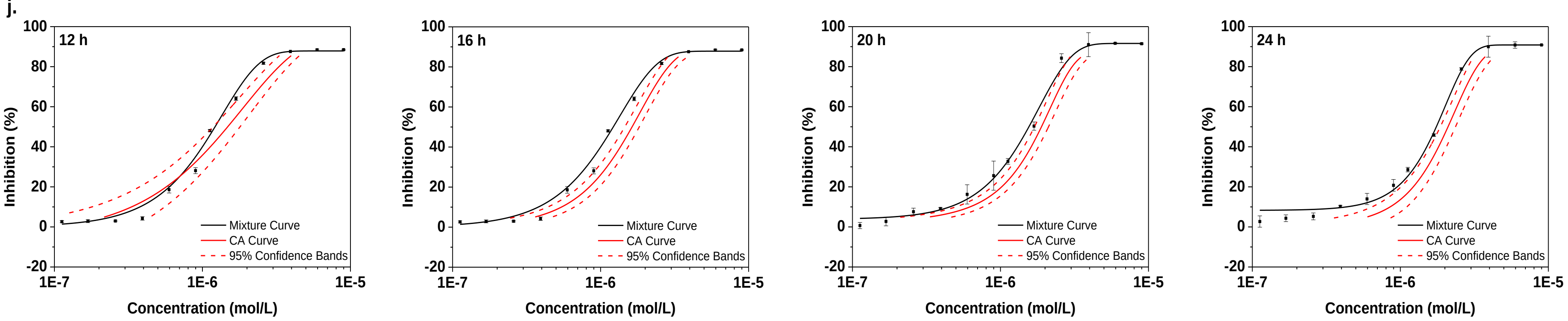

Figure S6. The CRCs for *E. coli* following exposure to the binary mixture of SAs and ERY at 12, 16, 20 and 24 h showing that they crossed the related CA curves with 95% confidence bands: (a) SD and ERY; (b) SDX and ERY; (c) SM and ERY; (d) SMM and ERY; (e) SMP and ERY; (f) SMR and ERY; (g) SMZ and ERY; (h) SPY and ERY; (i) SQ and ERY; and (j) SSZ and ERY.

Table S1. The primer sequences of *rrsG* and *sdiA*.

**The primer sequences**	***rrsG***	***sdiA***
Forward	TATTGCACAATGGGCGCAAG	GAGGATGGAGACCGCAGAA
Reverse	ACTTAACAAACCGCCTGCGT	TAATAACTAACCCACGCCTCA
